# CTLA-4 Protects against Angiotensin II-Induced Abdominal Aortic Aneurysm Formation in Mice

**DOI:** 10.1038/s41598-019-44523-6

**Published:** 2019-05-30

**Authors:** Hilman Zulkifli Amin, Naoto Sasaki, Tomoya Yamashita, Taiji Mizoguchi, Tomohiro Hayashi, Takuo Emoto, Takuya Matsumoto, Naofumi Yoshida, Tokiko Tabata, Sayo Horibe, Shoji Kawauchi, Yoshiyuki Rikitake, Ken-ichi Hirata

**Affiliations:** 1 Division of Cardiovascular Medicine, Department of Internal Medicine, Kobe University Graduate School of Medicine, Kobe, Japan; 2 Laboratory of Medical Pharmaceutics, Kobe Pharmaceutical University, Kobe, Japan; 3 Faculty of Medicine, Universitas Indonesia, Jakarta, Indonesia; 4 Education and Research Center for Clinical Pharmacy, Kobe Pharmaceutical University, Kobe, Japan

**Keywords:** Inflammation, Aneurysm

## Abstract

Vascular inflammation via T-cell-mediated immune responses has been shown to be critically involved in the pathogenesis of abdominal aortic aneurysm (AAA). T-cell coinhibitory molecule cytotoxic T-lymphocyte–associated antigen-4 (CTLA-4) is known to act as a potent negative regulator of immune responses. However, the role of this molecule in the development of AAA remains completely unknown. We determined the effects of CTLA-4 overexpression on experimental AAA. We continuously infused CTLA-4 transgenic (CTLA-4-Tg)/apolipoprotein E–deficient (*Apoe*^−/−^) mice or control *Apoe*^−/−^ mice fed a high-cholesterol diet with angiotensin II by implanting osmotic mini-pumps and evaluated the development of AAA. Ninety percent of angiotensin II-infused mice developed AAA, with 50% mortality because of aneurysm rupture. Overexpression of CTLA-4 significantly reduced the incidence (66%), mortality (26%), and diameter of AAA. These protective effects were associated with a decreased number of effector CD4^+^ T cells and the downregulated expression of costimulatory molecules CD80 and CD86, ligands for CTLA-4, on CD11c^+^ dendritic cells in lymphoid tissues. CTLA-4-Tg/*Apoe*^−/−^ mice had reduced accumulation of macrophages and CD4^+^ T cells, leading to attenuated aortic inflammation, preserved vessel integrity, and decreased susceptibility to AAA and aortic rupture. Our findings suggest T-cell coinhibitory molecule CTLA-4 as a novel therapeutic target for AAA.

## Introduction

Abdominal aortic aneurysm (AAA) remains an important cause of morbidity and mortality in developed countries^1^. However, none of the treatment approaches are effective to prevent AAA growth and rupture. Thus, it would be highly desirable to extensively investigate AAA pathophysiology and to develop new therapeutic options to prevent AAA.

Accumulating evidence suggests that chronic inflammation of the arterial wall is critically involved in the pathogenesis of AAA. Pathogenic innate and adaptive immune responses have been shown to evoke aortic inflammatory reactions and critically contribute to the development and rupture of experimental AAA^2^. Although important antigens responsible for driving AAA formation remain unidentified, after antigen presentation by antigen-presenting cells such as dendritic cells (DCs), naïve CD4^+^ T cells differentiate into different effector T cell (Teff) lineages such as T helper type 1 (Th_1_), T helper type 2, and T helper type 17 cells. Differentiated Teffs play a key role in provoking vascular inflammation and subsequent development of AAA, although the role of each helper T-cell subset in the pathogenesis of AAA is still controversial^2^. Previous experimental studies from several independent groups have demonstrated a protective role of forkhead box P3 (Foxp3)-expressing regulatory T cells (Tregs), which play an important role in dominant suppression of excessive immunoinflammatory reactions and maintenance of immune homeostasis^3^, in angiotensin II-induced experimental AAA^4,5^. Tipping the Treg/Teff balance toward Treg function by suppressing Teff responses and promoting Treg responses could be a feasible therapeutic approach for preventing AAA formation^5,6^.

There are two important signals from antigen-presenting cells required for naïve T cell activation. One is the signals from the T-cell receptor that is activated by interacting with antigenic peptide/major histocompatibility complex ligand on the antigen-presenting cells, which is essential for T cell activation. The other is the costimulatory signals provided by costimulatory molecules on antigen-presenting cells, which also play indispensable roles in enhancing or inhibiting activation of Teffs, depending on the type of costimulation. The T-cell costimulatory and coinhibitory pathways are crucial in modulating functions of Teffs and Tregs and their balance. Recent experimental studies using genetically modified mice or blocking antibodies have revealed that the costimulatory and coinhibitory pathways are critically involved in the pathogenesis of atherosclerosis^7^. Interactions of CD28 on T cells with B7 ligands CD80 and CD86 on antigen-presenting cells are the most important costimulatory pathway for T cell activation^8^. The CD80/CD86-CD28 costimulatory pathway has significant effects on the development of atherosclerosis^9,10^ and AAA^11^ by modulating the balance between Teffs and Tregs.

The coinhibitory molecule cytotoxic T-lymphocyte–associated antigen-4 (CTLA-4), specifically expressed in activated T cells and CD4^+^Foxp3^+^ Tregs, binds to CD80 and CD86 on antigen-presenting cells and negatively regulates T cell function. Our recent work using atherosclerosis-prone CTLA-4 transgenic (CTLA-4-Tg) mice demonstrated a protective role of this inhibitory molecule in the development of experimental atherosclerosis^12^. However, it is unknown whether CTLA-4 plays a protective role in the development of atherosclerosis-related cardiovascular diseases such as AAA. Considering immunoinflammatory mechanisms shared between AAA and atherosclerotic disease, we hypothesized that augmentation of CTLA-4 function would inhibit AAA formation. Here, using hypercholesterolemic CTLA-4-Tg mice constitutively expressing CTLA-4 on the cell surface and intracellularly in T cells^12,13^, we explored the role of CTLA-4 in the development of experimental AAA.

## Results

### Overexpression of CTLA-4 prevents the development of angiotensin II-induced AAA and reduces the mortality and severity in apolipoprotein E-deficient (*Apoe*^−/−^) mice

**Figure 1:**
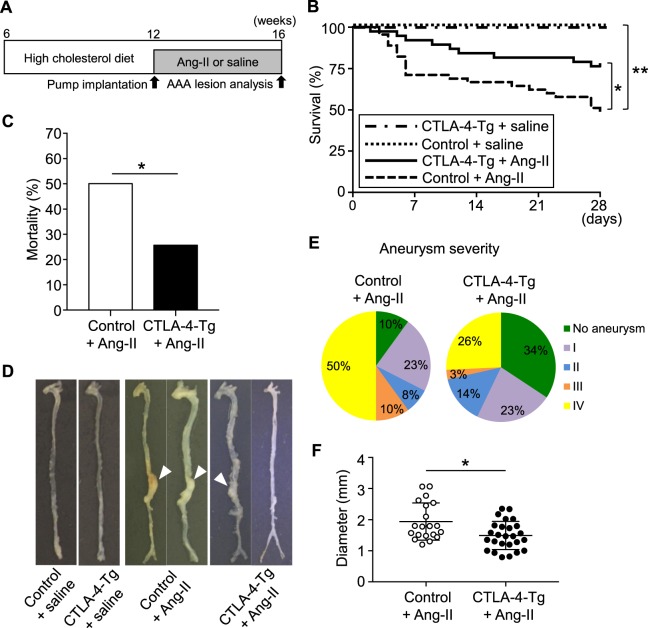
Overexpression of cytotoxic T lymphocyte-associated antigen-4 (CTLA-4) prevents the development of angiotensin II-induced abdominal aortic aneurysm (AAA) and reduces the mortality and severity in *Apoe*^−/−^ mice. (**A**) Experimental design. Twelve-week-old apolipoprotein E-deficient (*Apoe*^−/−^) mice or CTLA-4-Tg/*Apoe*^−/−^ mice fed a high-cholesterol diet were infused with angiotensin II or saline for 28 days and were euthanized at 16 weeks of age for evaluation of AAA formation. *Apoe*^−/−^ mice infused with angiotensin II or saline served as controls. (**B**) Kaplan-Meier curve shows survival rate in angiotensin II-infused *Apoe*^−/−^ (n = 40) and CTLA-4-Tg/*Apoe*^−/−^ (n = 35), or saline-infused *Apoe*^−/−^ (n = 5) and CTLA-4-Tg/*Apoe*^−/−^ (n = 5) mice. *P < 0.05; **P < 0.01; log-rank (Mantel-Cox) test. (**C**) Mortality due to AAA rupture in angiotensin II-infused *Apoe*^−/−^ (n = 40), CTLA-4-Tg/*Apoe*^−/−^ (n = 35) mice. *P < 0.05; chi-squared test. (**D**) Representative photographs displaying macroscopic features of aneurysms induced by angiotensin II. None of the animals infused with saline developed aneurysm. Arrowheads indicate abdominal aneurysms. (**E**) Severity of aneurysm in angiotensin II-infused *Apoe*^−/−^ (n = 40) and CTLA-4-Tg/*Apoe*^−/−^ (n = 35). (**F**) Maximal diameter of abdominal aorta in angiotensin II-infused *Apoe*^−/−^ (n = 20) and CTLA-4-Tg/*Apoe*^−/−^ (n = 26). Data points represent individual animals. Horizontal bars represent means. Error bars indicate s.d. *P < 0.05; Mann-Whitney *U*-test. Ang-II, angiotensin II.

To examine the effect of CTLA-4 overexpression in T cells on the development of AAA, we established hypercholesterolemic CTLA-4-Tg/*Apoe*^−/−^ mice expressing full-length CTLA-4 under the control of human CD2 promoter^12^ on atherosclerosis-prone background, and induced AAA by continuous angiotensin II infusion in these mice. At 16 weeks of age, the mice were euthanized and AAA formation was evaluated (Fig. 1A). CTLA-4 overexpression did not significantly affect body weight or plasma lipid profile (Supplemental Table 1). Angiotensin II infusion for 4 weeks led to a marked elevation in systolic blood pressure (SBP) in both 16-week-old *Apoe*^−/−^ and CTLA-4-Tg/*Apoe*^−/−^ mice, whereas there was no significant difference in SBP between the 2 groups (Supplemental Table 1). Ninety-three % of angiotensin II-infused *Apoe*^−/−^ mice developed AAA with 50% mortality due to an aneurysm rupture (Fig. 1B–E). On the other hand, we found a significant decrease in the incidence (66%, *P* = 0.0104) and mortality (26%, *P* = 0.031) of AAA in angiotensin II-infused CTLA-4-Tg/*Apoe*^−/−^ mice (Fig. 1B–E). In addition, we observed significantly decreased severity and a significant reduction in the diameter (18%, *P* = 0.011) of AAA in angiotensin II-infused CTLA-4-Tg/*Apoe*^−/−^ mice (Fig. 1E,F). Collectively, these data demonstrate the beneficial effects of CTLA-4 overexpression on AAA development and rupture.

### Overexpression of CTLA-4 limits elastin degradation and inflammatory cell recruitment into the aneurysmal lesions

**Figure 2:**
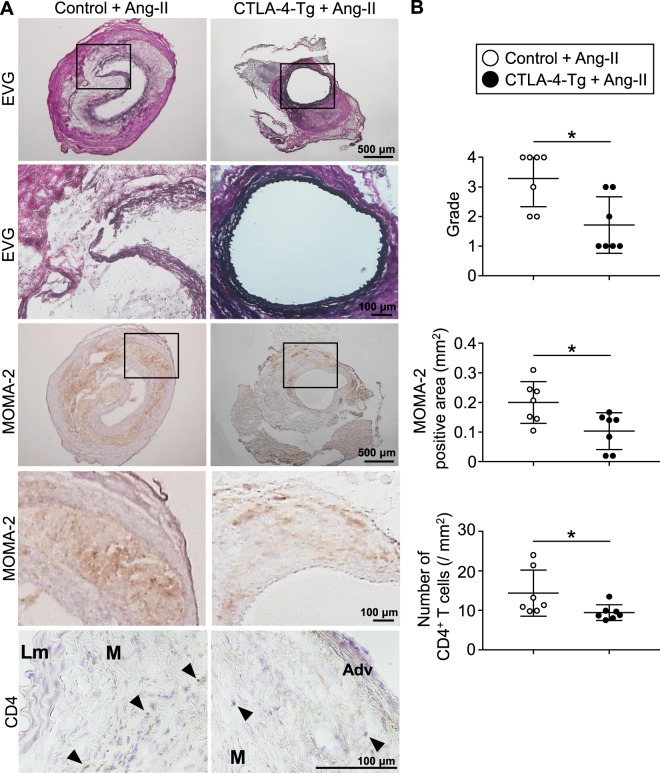
Overexpression of cytotoxic T lymphocyte-associated antigen-4 (CTLA-4) limits elastin degradation and the accumulation of macrophages and effector T cells in aneurysmal tissues. (**A**) Representative photomicrographs of elastin, MOMA-2, and CD4 staining in the abdominal aortic aneurysm lesions of angiotensin II-infused apolipoprotein E-deficient (*Apoe*^−/−^) and CTLA-4-Tg/*Apoe*^−/−^ mice. Boxed areas are expanded to show representative high-power fields in serial sections. Lm indicates lumen; M, media; Adv, adventitia. Arrowheads indicate CD4^+^ T cells. (**B**) Categorical score of elastin degradation and quantitative analyses of MOMA-2^+^ macrophages and CD4^+^ T cells in the aneurysmal lesions of angiotensin II-infused *Apoe*^−/−^ and CTLA-4-Tg/*Apoe*^−/−^ mice. n = 7 per group. Data points represent individual animals. Horizontal bars represent means. Error bars indicate s.d. *P < 0.05; Mann-Whitney *U*-test or unpaired *t*-test. Ang-II, angiotensin II.

Histological analysis of the aortic aneurysm tissues revealed that angiotensin II-infused CTLA-4-Tg/*Apoe*^−/−^ mice had more preserved elastin content compared with control mice (Fig. 2A,B). The aneurysmal lesions in angiotensin II-induced AAA are reported to have massive accumulation of inflammatory immune cells such as T cells and macrophages, which contributes to the development and rupture of AAA by inducing aortic inflammation^2^. By immunohistochemical studies of the aneurysmal lesions, we observed a significant reduction in the accumulation of these inflammatory cells into the aneurysmal lesions of angiotensin II-infused CTLA-4-Tg/*Apoe*^−/−^ mice compared with control mice (Fig. 2A,B). Together, these results suggest that overexpression of CTLA-4 attenuated inflammatory cell recruitment into the aneurysmal lesions and preserved vessel integrity, which may contribute to decreased susceptibility to AAA and aortic rupture.

### Effects of CTLA-4 overexpression on the development and activation of CD4^+^ T cells

**Figure 3:**
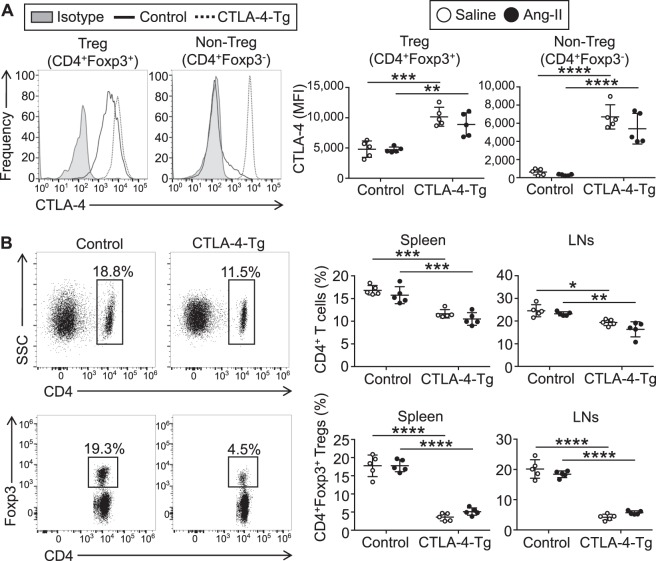
Overexpression of cytotoxic T lymphocyte-associated antigen-4 (CTLA-4) decreases the frequency of CD4^+^ T cells and CD4^+^ forkhead box P3 (Foxp3)^+^ regulatory T cells (Tregs) in angiotensin II-treated or saline-treated mice. Seven days after the pump implantation, lymphoid cells from spleen and lymph nodes (LNs) were prepared. Apolipoprotein E-deficient (*Apoe*^−/−^) mice infused with angiotensin II or saline served as controls. (**A**) Representative results of intracellular CTLA-4 expression in splenic CD4^+^Foxp3^+^ Tregs and CD4^+^Foxp3^−^ T cells of angiotensin II-infused *Apoe*^−/−^ and CTLA-4-Tg/*Apoe*^−/−^ mice assessed by flow cytometry. The expression levels of intracellular CTLA-4 were examined. The data are shown as mean fluorescence intensity (MFI). n = 5 per group. (**B**) Representative results of Foxp3 and CD4 expression in the spleen of angiotensin II-infused *Apoe*^−/−^ and CTLA-4-Tg/*Apoe*^−/−^ mice assessed by flow cytometry. The graphs represent the percentage of CD4^+^ T cells or CD4^+^Foxp3^+^ Tregs within spleen and LN cells or spleen and LN CD4^+^ population, respectively. n = 5 mice per group. Data points represent individual animals. Horizontal bars represent means. Error bars indicate s.d. *P < 0.05; **P < 0.01; ***P < 0.001; ****P < 0.0001; one-way ANOVA followed by Tukey’s post hoc test. Ang-II, angiotensin II; SSC, side scatter.

Next, we analyzed the changes in systemic immune responses and attempted to determine the mechanisms by which CTLA-4 overexpression prevents the development of AAA. Consistent with our previous report using the same CTLA-4-Tg/*Apoe*^−/−^ mice without angiotensin II treatment^12^, high expression levels of CTLA-4 were observed in the majority of CD4^+^Foxp3^−^ T cells in the spleen and lymph nodes (LNs) of angiotensin II-infused CTLA-4-Tg/*Apoe*^−/−^ mice, while only a minor proportion of CD4^+^Foxp3^−^ T cells expressed CTLA-4 in angiotensin II-infused *Apoe*^−/−^ mice (Fig. 3A). We found that CD4^+^Foxp3^+^ Tregs from both angiotensin II-infused *Apoe*^−/−^ and CTLA-4-Tg/*Apoe*^−/−^ mice highly expressed CTLA-4, which was further up-regulated in CD4^+^Foxp3^+^ Tregs from angiotensin II-infused CTLA-4-Tg/*Apoe*^−/−^ mice compared with those from angiotensin II-infused *Apoe*^−/−^ mice (Fig. 3A). The number of CD4^+^ T cells was lower in the LNs and spleen of angiotensin II-infused CTLA-4-Tg/*Apoe*^−/−^ mice than angiotensin II-infused *Apoe*^−/−^ mice (Fig. 3B), possibly due to their reduced proliferative capacity as described previously^12^. The frequency of CD4^+^Foxp3^+^ Tregs was markedly decreased in the LNs and spleen of angiotensin II-infused CTLA-4-Tg/*Apoe*^−/−^ mice (Fig. 3B), possibly due to their impaired development in the thymus as described previously^12^. There were no significant differences in the expression of typical Treg markers including CD25, CD103, and glucocorticoid-induced tumor necrosis factor receptor family-related gene/protein between the groups (Supplemental Fig. 1). Angiotensin II infusion did not significantly affect the numbers of CD4^+^ T cells or CD4^+^Foxp3^+^ Tregs or the expression of typical Treg markers in CD4^+^Foxp3^+^ Tregs. The effects of CTLA-4 overexpression on these immune cell numbers and functional markers were not significantly different between angiotensin II-treated and saline-treated mice. Neither angiotensin II infusion nor CTLA-4 overexpression affected significantly the numbers of other immune cell subsets such as CD8^+^ T cells, B cells, natural killer cells, natural killer T cells, Ly6C^high^ monocytes, and neutrophils in the spleen (Supplemental Fig. 2).

**Figure 4:**
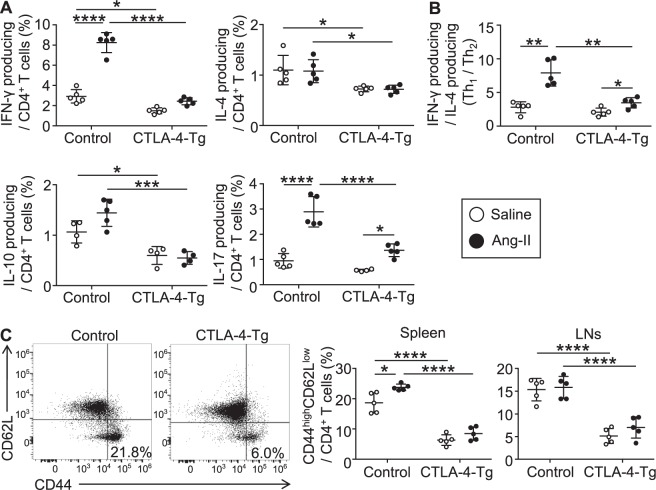
Overexpression of cytotoxic T lymphocyte-associated antigen-4 (CTLA-4) suppresses systemic T cell immune responses in angiotensin II-treated or saline-treated mice. Seven days after the pump implantation, lymphoid cells from spleen and lymph nodes (LNs) were prepared. Apolipoprotein E-deficient (*Apoe*^−/−^) mice infused with angiotensin II or saline served as controls. (**A**) The graphs represent the frequencies of interferon (IFN)-γ^+^, interleukin (IL)-4^+^, IL-10^+^, and IL-17^+^ CD4^+^ T cells in the spleen of each group. n = 5 per group. (**B**) The ratio of IFN-γ^+^ CD4^+^ T cells to IL-4^+^ CD4^+^ T cells was determined as T helper type 1 (Th_1_)/Th_2_ ratio. n = 5 per group. (**C**) Representative results of CD44 and CD62L expression in splenic CD4^+^ T cells of angiotensin II-infused *Apoe*^−/−^ and CTLA-4-Tg/*Apoe*^−/−^ mice assessed by flow cytometry. The graphs represent the percentage of CD44^high^CD62L^low^ effector T cells within the spleen and LN CD4^+^ population. n = 5 mice per group. Data points represent individual animals. Horizontal bars represent means. Error bars indicate s.d. *P < 0.05; **P < 0.01; ***P < 0.001; ****P < 0.0001; one-way ANOVA followed by Tukey’s post hoc test. Ang-II, angiotensin II.

To determine whether CTLA-4 overexpression affects T-cell responses such as cytokine secretion, we performed intracellular cytokine staining of splenic lymphocytes, and found that splenic interferon (IFN)-γ, interleukin (IL)-4, IL-10, and IL-17 producing CD4^+^ T cells were all significantly decreased in angiotensin II-infused CTLA-4-Tg/*Apoe*^−/−^ mice compared with angiotensin II-infused *Apoe*^−/−^ mice (Fig. 4A). Similar trends were observed in saline-infused mice (Fig. 4A). Notably, angiotensin II infusion led to a marked increase in IFN-γ or IL-17 producing CD4^+^ T cells and a shift of the Th_1_/Th_2_ balance (a ratio of IFN-γ^+^ CD4^+^ T cells to IL-4^+^ CD4^+^ T cells) toward a Th_1_ immune response in the spleen of *Apoe*^−/−^ mice (Fig. 4A,B). The angiotensin II-mediated increases in the numbers of IFN-γ or IL-17 producing CD4^+^ T cells and modulation of the Th_1_/Th_2_ balance tended to be attenuated by CTLA-4 overexpression (Fig. 4A,B). We further investigated the effect of CTLA-4 overexpression on T cell activation status by evaluating its activation markers in the peripheral LNs and spleen of *Apoe*^−/−^ or CTLA-4-Tg/*Apoe*^−/−^ mice. Angiotensin II infusion resulted in a marked increase in the proportion of CD44^high^CD62L^low^CD4^+^ T cells in the spleen but not in the LNs of *Apoe*^−/−^ mice (Fig. 4C). Notably, angiotensin II-dependent expansion of CD44^high^CD62L^low^CD4^+^ T cells was not observed in CTLA-4-Tg/*Apoe*^−/−^ mice (Fig. 4C). In line with the reduction in all the helper T cell subsets by CTLA-4 overexpression, we found a marked decrease in the proportion of CD44^high^CD62L^low^CD4^+^ Teffs in the spleen and LNs of angiotensin II-infused or saline-infused CTLA-4-Tg/*Apoe*^−/−^ mice compared with angiotensin II-infused or saline-infused *Apoe*^−/−^ mice, respectively (Fig. 4C). In addition, a decrease in the proportion of CD44^high^CD62L^low^CD4^+^ T cells in the para-aortic LNs was also observed following CTLA-4 overexpression (Supplemental Fig. 3). Collectively, these results suggest that CTLA-4 overexpression inhibits CD4^+^ T-cell activation and their accumulation in the para-aortic LNs and aneurysmal lesions of angiotensin II-infused hypercholesterolemic mice, leading to suppression of pathogenic immunoinflammatory responses and AAA development.

### Overexpression of CTLA-4 limits maturation of DCs

**Figure 5:**
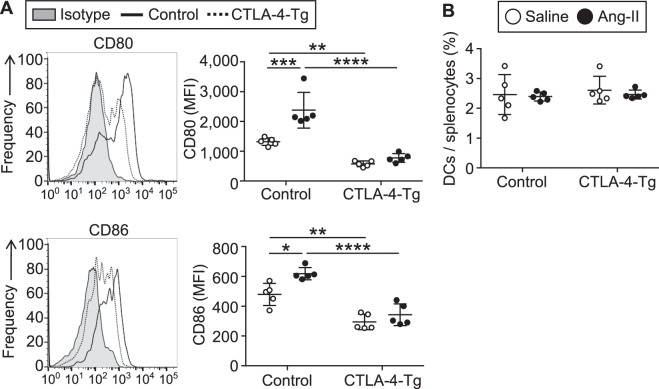
Overexpression of cytotoxic T lymphocyte-associated antigen-4 (CTLA-4) regulates dendritic cell (DC) activation in the mice infused with angiotensin II or saline. Seven days after the pump implantation, lymphoid cells from spleen and lymph nodes (LNs) were prepared. Apolipoprotein E-deficient (*Apoe*^−/−^) mice infused with angiotensin II or saline served as controls. (**A**) Representative results of CD80 and CD86 expression in splenic CD11c^+^ major histocompatibility complex (MHC)-II^+^ DCs of angiotensin II-infused *Apoe*^−/−^ and CTLA-4-Tg/*Apoe*^−/−^ mice assessed by flow cytometry. The expression levels of CD80 and CD86 were analyzed gating on splenic CD11c^+^MHC-II^+^ DCs. The data are shown as mean fluorescence intensity (MFI). n = 5 per group. (**B**) The graph represents the percentage of CD11c^+^MHC-II^+^ DCs within splenocytes. n = 5 per group. Data points represent individual animals. Horizontal bars represent means. Error bars indicate s.d. *P < 0.05; **P < 0.01; ***P < 0.001; ****P < 0.0001; one-way ANOVA followed by Tukey’s post hoc test. Ang-II, angiotensin II.

Costimulatory molecules such as CD80 and CD86 are mainly expressed on DCs, bind to CD28 on T cells and induce T cell activation^8^. We found that CD11c^+^ DCs from angiotensin II-infused *Apoe*^−/−^ mice expressed higher levels of CD80 and CD86 compared with those from saline-infused *Apoe*^−/−^ mice (Fig. 5A). CTLA-4 overexpression significantly decreased CD80 and CD86 expression in angiotensin II-infused *Apoe*^−/−^ mice (Fig. 5A), indicating suppression of DC maturation mediated by binding of overexpressed CTLA-4 to CD80/CD86 on DCs. Notably, the angiotensin II-dependent increase in CD80 and CD86 expression on DCs was completely abrogated by CTLA-4 overexpression (Fig. 5A). CTLA-4 overexpression did not change the proportion of CD11c^+^ DCs in the spleen (Fig. 5B). These results suggest that the upregulation of CD80 and CD86 expression on DCs may be critical for augmenting pathogenic T cell immune responses and AAA formation following angiotensin II infusion, and that CTLA-4 overexpression potently blunts the angiotensin II-induced DC maturation and subsequent pathogenic T cell immune responses.

### CTLA-4-mediated prevention of the development and rupture of angiotensin II-induced AAA is associated with reduced aortic immunoinflammatory responses

**Figure 6:**
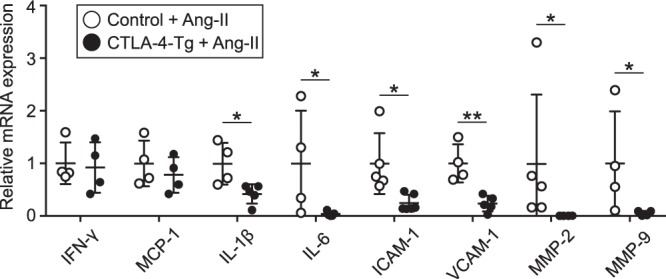
Overexpression of cytotoxic T lymphocyte-associated antigen-4 (CTLA-4) suppresses immunoinflammatory responses in aneurysmal tissues. Seven days after the pump implantation, angiotensin II-infused apolipoprotein E-deficient (*Apoe*^−/−^) and CTLA-4-Tg/*Apoe*^−/−^ mice were euthanized and total RNA was extracted from the supra-renal aortas. Messenger RNA expression of proinflammatory cytokines (interferon (IFN)-γ, interleukin (IL)-1β, IL-6), chemokine monocyte chemoattractant protein (MCP)-1, adhesion molecules (intercellular adhesion molecule (ICAM)-1, vascular cell adhesion molecule (VCAM)-1), and matrix metalloproteinase (MMPs) (MMP-2, MMP-9) was quantified by quantitative real-time reverse transcription PCR and normalized to GAPDH. n = 4 to 6 per group. Data points represent individual animals. Horizontal bars represent means. Error bars indicate s.d. *P < 0.05; **P < 0.01; Mann-Whitney *U*-test. Ang-II, angiotensin II.

To reveal the mechanisms of decreased development and rupture of angiotensin II-induced AAA, we examined mRNA expression of immunoinflammatory molecules in suprarenal aortas by quantitative reverse transcription polymerase chain reaction. Notably, proinflammatory cytokines (IL-1β and IL-6), adhesion molecules (intercellular adhesion molecule (ICAM)-1 and vascular cell adhesion molecule (VCAM)-1), and matrix metalloproteinase (MMP)-2 and MMP-9 were markedly reduced in angiotensin II-infused CTLA-4-Tg/*Apoe*^−/−^ mice compared with angiotensin II-infused *Apoe*^−/−^ mice (Fig. 6). There were no differences in the mRNA expression of proinflammatory IFN-γ and monocyte chemoattractant protein (MCP)-1 between angiotensin II-infused CTLA-4-Tg/*Apoe*^−/−^ mice and *Apoe*^−/−^ mice (Fig. 6). Taken together, these data suggest that CTLA-4 overexpression suppresses inflammatory cell migration into the aneurysmal lesions and aortic inflammation and preserved vessel integrity, which may be crucially involved in the protection against AAA development and aortic rupture.

## Discussion

Treatment approaches such as management of lifestyle-associated risk factors is not effective to prevent the development of AAA, a life-threatening immunoinflammatory aortic disease. Although relatively high-risk surgical therapy is performed for large aneurysms, we do not have effective medical therapies against AAA at the initial stages, which is due to insufficient information about the pathogenesis of AAA. Compelling evidence indicates that chronic immunoinflammatory responses mediated by adaptive immune cells such as T cells are detrimental for the development of AAA^2^. In the present study, using hypercholesterolemic mice constitutively expressing T-cell coinhibitory molecule CTLA-4, which is known to play a critical role for negatively regulating T cell immune responses, in T cells, we have clearly demonstrated, for the first time to our knowledge, that CTLA-4 overexpression in T cells effectively prevents AAA formation by suppressing systemic and aortic immunoinflammatory responses in angiotensin II-infused hypercholesterolemic mice. Thus, our data suggest that CTLA-4 could be a novel therapeutic target for AAA.

It is now evident that innate and adaptive immune systems, in particular T-cell-mediated immune responses, crucially contribute to driving inflammation involved in the pathogenesis of AAA and atherosclerotic disease. However, the role of each Teff subset in these diseases may vary depending on species, animal models, and experimental conditions. In addition, experimental and clinical evidence shows many differences in the contribution of various Teff immune responses including the Th_1_/Th_2_ balance to disease development and progression between AAA and atherosclerotic disease^2^. Notably, we found here that the protective effects of CTLA-4 in AAA were associated with attenuation of the angiotensin II-mediated shift of the Th_1_/Th_2_ balance toward a Th_1_ immune response, as well as suppression of all helper T-cell-mediated immune responses such as Th_1_, Th_2_, and Th_17_ immune responses. Considering that the Th_1_ immune response promotes atherosclerosis, our findings suggest that intervention to enhance CTLA-4 function could be an attractive strategy to treat patients with AAA who are at high risk of atherosclerotic cardiovascular disease.

T-cell costimulatory or coinhibitory pathways are known to play critical roles in enhancing or inhibiting activation of T cells, respectively. The important costimulatory pathway involving the interactions of B7 ligands CD80 and CD86 on DCs with CD28 on T cells has been suggested to play a dominant role in the modulation of T cell functions and atherosclerosis^7^. A recent study showed that normocholesterolemic CD80^−/−^/CD86^−/−^ or CD28^−/−^ mice exhibited a marked decrease in Tregs and increased incidence and mortality of angiotensin II-induced AAA^11^. Of note, this study also showed that reconstitution of CD28^−/−^ mice with wild-type Tregs protected the mice from the development and rupture of AAA, providing direct evidence that impaired Treg function, but not modulation of Teff immune responses, is a central mechanism for the exacerbation of AAA formation under the condition of the CD80/CD86-CD28 pathway blockade. Although a possible role of the coinhibitory molecule CTLA-4 in the protection against atherosclerosis has been suggested^12,14^, its role in the development of atherosclerosis-related cardiovascular diseases such as AAA remains completely unknown so far. In the present study, we provide first evidence that augmentation of CTLA-4 function protects against AAA formation by regulating immunoinflammatory responses. These CTLA-4-dependent favorable effects may involve several mechanisms including cell intrinsic or cell extrinsic pathways. As a cell intrinsic mechanism, T cells receive a negative signal through its cytoplasmic tail by the interactions of CD80 and CD86 on DCs with CTLA-4 on T cells, and play a pivotal role in the modulation of T cell receptor signaling and its functions^15^. Our previous study using atherosclerotic CTLA-4-Tg/*Apoe*^−/−^ mice revealed that CTLA-4 transgenic CD4^+^ T cells have a lower proliferative capacity than wild-type CD4^+^ T cells upon anti-CD3 antibody stimulation^12^, indicating the possible involvement of the cell intrinsic mechanism for CTLA-4-mediated protection from AAA as well as atherosclerosis. Experimental evidence indicates that cell extrinsic pathways such as down-regulation of CD80 and CD86 on DCs are critical for CTLA-4-mediated immune regulation in several autoimmune settings^15^. Consistent with this idea, we observed a remarkable decrease in the CD80 and CD86 expression on DCs in angiotensin II-infused CTLA-4-Tg/*Apoe*^−/−^ mice compared with angiotensin II-infused *Apoe*^−/−^ mice, indicating that CTLA-4 transgenic T cells have an ability to regulate DC maturation in a cell extrinsic manner. We found a marked augmentation of Teff immune responses following angiotensin II infusion, which was associated with up-regulation of CD80 and CD86 expression on DCs, indicating that angiotensin II may augment Teff immune responses partly via inducing DC maturation. Taken together with our findings of the marked down-regulation of CD80 and CD86 expression on DCs in CTLA-4-Tg/*Apoe*^−/−^ mice, our data suggest that stimulation of DC maturation is an important mechanism for the CTLA-4-mediated protection against angiotensin II-induced AAA formation. CTLA-4 is known to have a stronger binding affinity than CD28 for their common ligands CD80 and CD86 and thereby reduces CD28 signals. Thus, as another cell extrinsic mechanism, overexpressed CTLA-4 on T cells may possibly act as a blocker for the CD80/CD86-CD28 pathway, leading to the inhibition of Teff immune responses and AAA development.

A large body of experimental evidence indicates that the dysregulated balance between pro-inflammatory Teffs and anti-inflammatory Tregs contributes to the development and progression of AAA^4,5,11^ and atherosclerotic disease^10,16,17^. Recent studies suggest that augmentation of the Treg/Teff ratio, by regulating Teff responses or promoting Treg responses, could be a feasible therapeutic approach for AAA^5,6^ as well as atherosclerosis^18–20^. Our findings of attenuated Teff immune responses and AAA development mediated by CTLA-4 overexpression does not appear to be due to modulation of Treg responses, because angiotensin II-infused CTLA-4-Tg/*Apoe*^−/−^ mice had a markedly reduced number of CD4^+^Foxp3^+^ Tregs compared with angiotensin II-infused *Apoe*^−/−^ mice. Notably, we found that anti-inflammatory IL-10 production from CD4^+^ T cells was markedly suppressed in angiotensin II-infused CTLA-4-Tg/*Apoe*^−/−^ mice, which may be partly explained by decreased numbers of CD4^+^Foxp3^+^ Tregs. It is possible that CTLA-4-mediated inhibition of proinflammatory Teff immune responses may overwhelm the detrimental effects of decreased Treg numbers and IL-10 production in our CTLA-4-Tg/*Apoe*^−/−^ mice.

Recent experimental evidence suggests that Teff immune responses are critically involved in the pathogenesis of hypertension^21^. Genetic or antibody-mediated abrogation of the costimulatory CD80/CD86-CD28 pathway prevents T-cell activation, vascular inflammation, and hypertensive response in various hypertensive murine models^22^, suggesting that inhibition of the costimulatory pathway may have therapeutic benefit for the treatment of this disease. Unfortunately, in our study, CTLA-4-dependent modulation of the CD80/CD86-CD28 pathway was not able to prevent the angiotensin II-induced hypertension. Of note, we found a marked decrease in the frequency and number of CD4^+^Foxp3^+^ Tregs in CTLA-4-Tg/*Apoe*^−/−^ mice. Because CD4^+^Foxp3^+^ Tregs have been shown to play a protective role in various murine models of hypertension^21^, no effect of CTLA-4 overexpression on systolic blood pressure seems to be because of attenuated Treg immune responses in our hypertensive mouse model.

The results of this study have important clinical implications. CTLA-4-Ig, a soluble fusion protein consisting of the extracellular CTLA-4 portion, mimics CTLA-4 and has been shown to be effective for treating patients with rheumatoid arthritis^15^. Rheumatoid arthritis has been shown to be linked to an increased risk of atherosclerotic cardiovascular disease^23^ and AAA^24^. Interestingly, a recent multidatabase cohort study has reported that treatment with CTLA-4-Ig was associated with a modestly reduced cardiovascular risk compared with tumor necrosis factor inhibitor treatment in rheumatoid arthritis patients with diabetes^25^. In consideration of our findings of the protective role of CTLA-4 in AAA formation, it will be of great interest to test the hypothesis that an immunomodulatory approach such as costimulatory blockade by CTLA-4-Ig would be effective for preventing the development of atherosclerotic cardiovascular disease and AAA in patients with rheumatoid arthritis. However, because activation of the coinhibitory pathways may have potential adverse effects such as general immunosuppression and exacerbation of infectious diseases, careful management is needed for application to clinical situations.

We provide evidence that the coinhibitory molecule CTLA-4 plays a critical role in limiting the development of angiotensin II-induced AAA and reducing the mortality by suppressing immunoinflammatory responses in the aortic aneurysmal lesions. Although further investigation will be needed before extrapolation of our experimental data to clinical settings, our findings identify CTLA-4 as an attractive therapeutic target for preventing AAA.

## Methods

### Animals and experimental design

CTLA-4-Tg/*Apoe*^−/−^ mice on a C57BL/6 background are previously described^12^. Six-week-old male mice were fed a high-cholesterol diet containing 0.2% cholesterol and 21% fat (CLEA, Tokyo, Japan) and water ad libitum. Twelve-week-old mice were infused with angiotensin II (1000 ng/kg/min, Sigma, St Louis, MO) or saline (sham group) for 7 or 28 days by implanting ALZET mini-osmotic pumps (Model 2004; DURECT Corp, Cupertino, CA) under anesthesia as described previously^26^. At 16 weeks of age, mice were euthanized by cervical dislocation under anesthesia for evaluating of AAA formation. We performed necropsy in all dead mice and found the formation of blood clots in their abdominal aortas that indicates the abdominal aortic rupture event. Some of the dead mice also showed evidence of thoracic aortic rupture. All dead mice were included in the analyses of mortality, incidence, and severity of AAA. We housed mice in specific pathogen-free animal facilities. All animal experiments were approved by the Committee on the Ethics of Animal Experiments of Kobe University Graduate School of Medicine (Permit Number: P120101) and by the Animal Care Committee of Kobe Pharmaceutical University (Permit Numbers: 2016-049, 2017-024, 2018-003) and conform to the NIH guidelines.

### Blood pressure measurement

SBP was measured by noninvasive tail-cuff method (BP-98 Softron, Tokyo, Japan) as described previously^5^. The SBP was measured at least five times at baseline and 4 weeks after angiotensin II pump implantation. The mean SBP for each group was determined by averaging the SBP of each mouse included in that group. The data from one day measurement of each time point were used.

### Assessment of biochemical parameters

After overnight fasting, blood was collected by the cardiac puncture under anesthesia. Plasma was obtained through centrifugation and stored at −80 °C until measurement. Concentrations of plasma total cholesterol and triglyceride were determined enzymatically using an automated chemistry analyzer (SRL, Tokyo, Japan).

### Morphological analysis of AAA

Aortic diameters and AAA incidence were determined as described previously^26^. For morphological analyses, aortas were perfused with saline and fixed in 10% buffered formalin. The maximum external aortic diameters were measured using ImageJ (National Institutes of Health, Bethesda, MD). Aneurysm incidence was quantified on the basis of a definition of an external suprarenal aorta width that was increased by 50% or more compared to saline-infused mice. We used a previously described classification system^5^ to categorize the morphological severity of the aneurysms: no aneurysm, type I (a discernable dilation that is 1.5 to 2 times the diameter of a normal abdominal aorta), type II (a single large dilation that is more than 2 times the diameter of a normal abdominal aorta), type III (multiple dilations generally extending proximal to the suprarenal region), type IV (death due to aneurysmal rupture).

### Histological and immunohistochemical analysis of aneurysmal lesions

Mice were anesthetized and the aorta was perfused with saline. The AAA lesions were cut and embedded in OCT compounds (Tissue-Tek; Sakura Finetek, Tokyo, Japan), and cross-sections (10 μm) were prepared. For the determination of elastin degradation, we performed Elastica van Gieson staining and used a standard score for the grades of elastin degradation as described previously^5^. Two sections were analyzed in each mouse and grading score was determined by calculating the average grade of the sections. The grades were defined as follows: grade 1, no degradation; grade 2, mild elastin degradation; grade 3, severe elastin degradation; grade 4, aortic rupture. Immunohistochemistry was performed on acetone-fixed or formalin-fixed cryosections (10 μm) of the maximum AAA lesions using antibodies to identify macrophages (MOMA-2, 1:400; BMA Biomedicals) and T cells (CD4, 1:100; BD Biosciences), followed by detection with biotinylated secondary antibodies and streptavidin-horseradish peroxidase. The appropriate fixation reagent depending on the primary antibodies was used. Stained sections were digitally captured using an All-in-one Type Fluorescence Microscope (BZ-8000; Keyence, Osaka, Japan) and the stained area was calculated. Two consecutive sections of AAA lesions with the maximum size were analyzed in each mouse and the average values were used for statistical analysis. Quantitative analysis of CD4^+^ T cells of the AAA lesion was performed by counting the positive-stained cells, which were divided by total area of each cross section.

### Flow cytometry

For fluorescent-activated cell sorter analysis of lymphoid tissues, inguinal, axillary, and para-aortic LN cells and splenocytes were isolated and stained in PBS containing 2% fetal calf serum. Several lymph nodes that located just beside the abdominal aorta or near the iliac bifurcation were defined as para-aortic LNs. Flow cytometric analysis was performed by Attune Acoustic Focusing Cytometer (Life Technologies) or FACSAria III (BD Biosciences) using FlowJo software (Tree Star). The antibodies used were as follows; anti-CD4 PECy7 (clone RM4-5; BD Biosciences), anti-CD4 APCy7 (clone GK1.5; BD Biosciences), anti-CD8 (clone 53-6.7; BD Biosciences), anti-CD25 (clone PC61; BD Biosciences), anti-CD44 (clone IM7; BD Biosciences), anti-CD62L (clone MEL-14; BD Biosciences), anti-CD103 (clone M290; BD Biosciences), anti-GITR (clone DTA1; BD Biosciences), anti-CTLA-4 (clone UC10; BD Biosciences), ani-Foxp3 (clone FJK-16s; eBioscience), anti-CD11b (clone M1/70; BD Biosciences), anti-CD11c (clone HL3; BD Biosciences), anti-CD80 (clone 16-10A1; BD Biosciences), anti-CD86 (clone GL1; BD Biosciences), anti-B220 (clone RA3-6B2; BD Biosciences), anti-NK1.1 (clone PK136; BD Biosciences), anti-Ly6C (clone AL-21; BD Biosciences), anti-Ly6G (clone 1A8; BD Biosciences), anti-IL-4, (clone BVD4-1D11; eBioscience), anti-IL-10 (clone JES5-16E3; eBioscience), anti-IL-17 (clone 17-B7; BD Bioscience), anti-IFN-γ (cloneXMG1.2; eBioscience), and isotype matched control antibodies. Intracellular staining of Foxp3 was performed using the Foxp3 staining buffer set (eBioscience) according to the manufacturer’s instructions. All staining procedure was performed after blocking Fc receptor with anti-CD16/CD32 (clone 2.4G2; BD Bioscience). Surface stainings were performed according to standard procedures at a density of 5–10 × 10^5^ cells per 50 μL, and volumes were scaled up accordingly.

### Intracellular cytokine staining

Splenocytes were stimulated with 20 ng/ml phorbol 12-myristate 13-acetate (Sigma) and 1 mmol/L ionomycin (Sigma) for 5 hours in the presence of Brefeldin A (eBioscience). After staining for surface antigens, intracellular cytokine staining was performed using an intracellular cytokine staining kit (BD Biosciences) and anti-cytokine antibodies according to the manufacturer’s instructions.

### Real-time reverse transcription (RT)-PCR analysis

Total RNA was extracted from aortas after perfusion with RNA later (Life Technologies) using TRIzol reagent (Life Technologies). For RT, a PrimeScript RT reagent Kit (Takara, Shiga, Japan) was used. Quantitative PCR was performed as described previously^20^ using a SYBR Premix Ex Taq (Takara) and a StepOnePlus Real-Time PCR System (Thermo Fisher Scientific) according to the manufacturer’s protocol. The primer sequences are shown in Supplemental Table 2. Amplification reactions were performed in duplicate and fluorescence curves were analyzed with included software. GAPDH was used as an endogenous control reference.

### Statistical analysis

Unpaired *t*-test or Mann-Whitney *U*-test was used to detect significant differences among 2 groups for normally distributed data or non-normally distributed data, respectively. The SBP data obtained before and after angiotensin II infusion in the same group were compared with paired *t*-test. One-way ANOVA followed by Tukey’s post hoc test was used to detect significant differences among 4 groups. Kaplan-Meier survival curves were constructed and analyzed using log-rank (Mantel-Cox) test. Incidence and mortality of AAA were analyzed using chi-squared test. Severity of AAA was analyzed using Fisher exact test. A value of *P* < 0.05 was considered statistically significant. For statistical analysis, GraphPad Prism version 7.0 (GraphPad Software) and EZR version 1.37^27^ were used.

## Supplementary information

Supplemental figures and tables

## Data Availability

All data generated or analyzed during this study are available from the corresponding author on reasonable request.
