# Supplementary material for: CTLA-4 Protects against Angiotensin II-Induced Abdominal Aortic Aneurysm Formation in Mice
Source: Sci Rep. 2019 May 30;9:8065. doi: 10.1038/s41598-019-44523-6 (PMC6542846; doi:10.1038/s41598-019-44523-6)
Supplement: Supplementary file 1 — Supplemental figures and tables [file 41598_2019_44523_MOESM1_ESM.pdf]

Supplemental Material

CTLA-4 Protects against Angiotensin II-Induced Abdominal Aortic Aneurysm Formation in Mice

Hilman Zulkifli Amin[1,2,3], Naoto Sasaki[1,2], Tomoya Yamashita[1], Taiji Mizoguchi[1,2], Tomohiro Hayashi[1], Takuo Emoto[1], Takuya Matsumoto[1], Naofumi Yoshida[1], Tokiko Tabata[1], Sayo Horibe[2], Shoji Kawauchi[4], Yoshiyuki Rikitake[2], Ken-ichi Hirata[1]

[1]Division of Cardiovascular Medicine, Department of Internal Medicine,
Kobe University Graduate School of Medicine, Kobe, Japan
[2]Laboratory of Medical Pharmaceutics, Kobe Pharmaceutical University, Kobe, Japan
[3]Faculty of Medicine, Universitas Indonesia, Jakarta, Indonesia
[4]Education and Research Center for Clinical Pharmacy, Kobe Pharmaceutical University, Kobe, Japan

Address correspondence to:
Naoto Sasaki, MD, PhD. E-mail: n-sasaki@kobepharma-u.ac.jp
Laboratory of Medical Pharmaceutics, Kobe Pharmaceutical University,
4-19-1, Motoyamakita-machi, Higashinada-ku, Kobe, 658-8558, Japan
Telephone number; +81-78-441-7579, FAX number; +81-78-441-7579

Supplemental Table 1

Parameters	Control	CTLA-4-Tg
Body weight, g	27.9 ± 1.6 (n=15)	28.7 ± 1.5 (n=15)
SBP (before), mmHg	103.5 ± 6.6 (n=15)	105.4 ± 4.7 (n=15)
SBP (after), mmHg	134.0 ± 14.1* (n=15)	128.7 ± 25.3* (n=15)
Total-cholesterol, mg/dl	826.3 ± 284.3 (n=19)	815.4 ± 163.9 (n=20)
Triglycerides, mg/dl	45.0 ± 23.6 (n=19)	50.2 ± 26.2 (n=20)

Body weight, SBP, and plasma lipid profile, in 16-week-old control apolipoprotein E-deficient (*Apoe*$^{-/-}$) and cytotoxic T lymphocyte-associated antigen-4 (CTLA-4)-Tg/*Apoe*$^{-/-}$ mice. Data are expressed as mean±SD. SBP indicates systolic blood pressure; SBP (before), SBP before angiotensin II infusion; SBP (after), SBP after angiotensin II infusion for 4 weeks. $^{*}P<0.05$ vs SBP (before) in the same group; paired *t*-test. There are no statistically significant differences in all parameters between angiotensin II-infused *Apoe*$^{-/-}$ and CTLA-4-Tg/*Apoe*$^{-/-}$ mice.

Supplemental Table 2

Genes	Forward primers	Reverse primers
Ifng	CGGCACAGTCATTGAAAGCCTA	GTTGCTGATGGCCTGATTGCT
Ccl2	GCATCCACGTGTTGGCTCA	CTCCAGCCTACTCATTGGGATCA
Il1b	TCCAGGATGAGGACATGAGCAC	GAACGTCACACACCAGCAGGTTA
Il6	CCACTTCACAAGTCGGAGGCTTTA	GCAAGTGCATCATCGTTGTTCATAC
Icam1	CAATTCACACTGAATGCCAGCTC	CAAGCAAGTCCGTCTCGTCCA
Vcam1	TGCCGGCATATACGAGTGTGA	CCCGATGGCAGGTATTACCAAG
Mmp2	GATAACCTGGATGCCGTCGTG	CTTCACGCTCTTGAGACTTTGGTTC
Mmp9	GCCCTGGAACTCACACGACA	TTGGAAACTCACACGCCAGAAG
Gapdh	TGTGTCCGTCGTGGATCTGA	TTGCTGTTGAAGTCGCAGGAG

Primer sequences.

Supplemental Figure 1

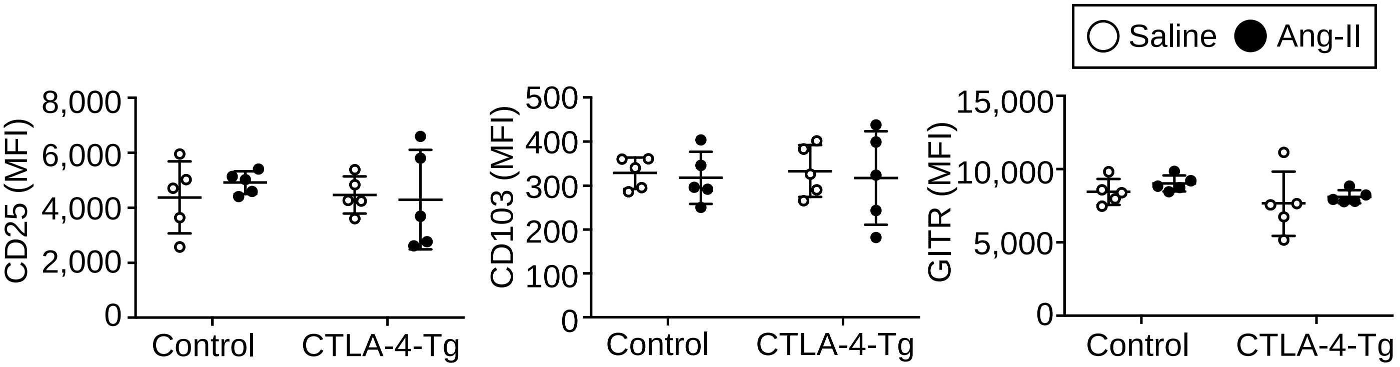

Supplemental Figure 1. The effect of cytotoxic T lymphocyte-associated antigen-4 (CTLA-4) overexpression on Treg-associated markers. Seven days after the pump implantation, apolipoprotein E-deficient (*Apoe*$^{-/-}$) and CTLA-4-Tg/*Apoe*$^{-/-}$ mice with or without angiotensin II infusion were euthanized and lymphoid cells from spleen were prepared. *Apoe*$^{-/-}$ mice infused with angiotensin II or saline served as controls. The expression levels of Treg-associated markers were analyzed gating on splenic $CD4^{+}Foxp3^{+}$ Tregs. Histograms show mean fluorescence intensity (MFI). n=5 per group. Data points represent individual animals. Horizontal bars represent means. Error bars indicate s.d. Ang-II, angiotensin II; GITR, glucocorticoid-induced tumor necrosis factor receptor family-related gene/protein.

Supplemental Figure 2

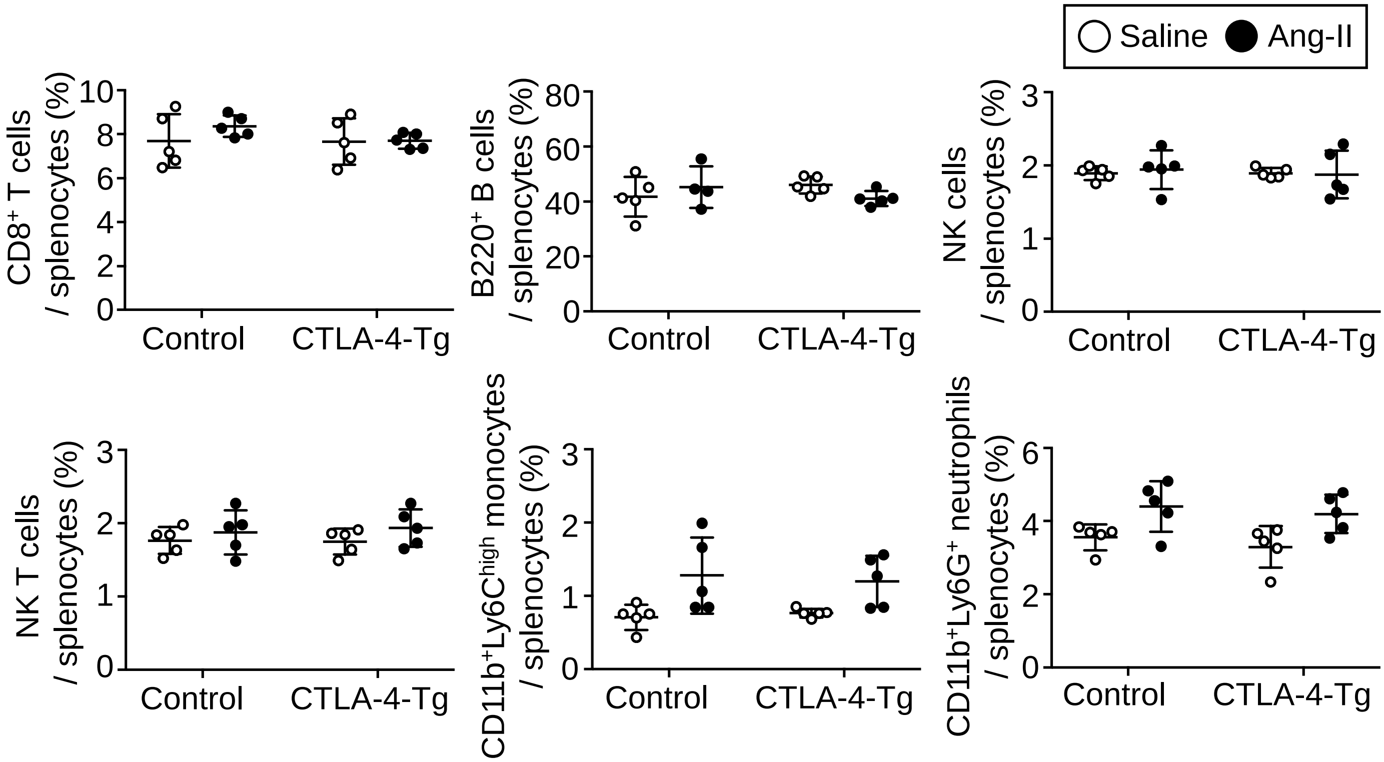

Supplemental Figure 2. Overexpression of cytotoxic T lymphocyte-associated antigen-4 (CTLA-4) does not affect immune cells other than $CD4^+$ T cells in spleen. Seven days after the pump implantation, apolipoprotein E-deficient (*Apoe*$^{-/-}$) and CTLA-4-Tg/*Apoe*$^{-/-}$ mice with angiotensin II or saline infusion were euthanized and lymphoid cells from spleen were prepared. *Apoe*$^{-/-}$ mice infused with angiotensin II or saline served as controls. Percentages of splenic $CD8^+$ T cells, $B220^+$ B cells, natural killer (NK) cells, NK T cells, $CD11b^+Ly6C^{high}$ monocytes, $CD11b^+Ly6G^+$ neutrophils were determined by flow cytometry. n=4 to 5 per group. Data points represent individual animals. Horizontal bars represent means. Error bars indicate s.d. Ang-II, angiotensin II.

Supplemental Figure 3

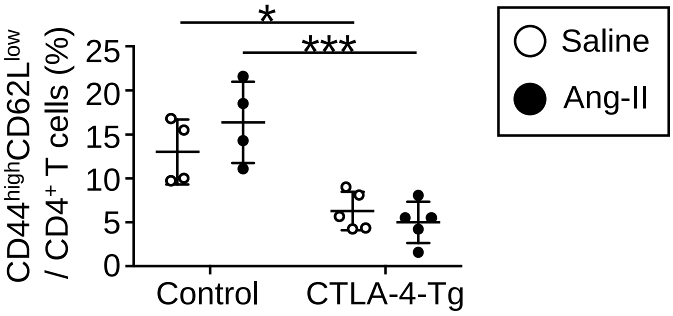

Supplemental Figure 3. Overexpression of cytotoxic T lymphocyte-associated antigen-4 (CTLA-4) suppresses Teff responses in the para-aortic lymph nodes (LNs) of angiotensin II-infused or saline-infused mice. Seven days after the pump implantation, lymphoid cells from para-aortic LNs were prepared. Apolipoprotein E-deficient (*Apoe*$^{-/-}$) mice infused with angiotensin II or saline served as controls. The graph represents the percentage of CD44^{high}CD62L^{low} effector T cells within the CD4^{+} population assessed by flow cytometry. n=4 to 5 per group. Data points represent individual animals. Horizontal bars represent means. Error bars indicate s.d. $^{*}P<0.05$; $^{***}P<0.001$; one-way ANOVA followed by Tukey's post hoc test. Ang-II, angiotensin II.
